# Supplementary material for: hBN Flake Embedded Al2O3 Thin Film for Flexible Moisture Barrier
Source: Materials (Basel). 2021 Dec 1;14(23):7373. doi: 10.3390/ma14237373 (PMC8658176; doi:10.3390/ma14237373)
Supplement: Supplementary file 1 [file materials-14-07373-s001.zip › materials-1455480-supplementary.pdf]

materials

MDPI

Supplementary Material

hBN Flake Embedded Al_2O_3 Thin Film for Flexible Moisture Barrier

Wonseok Jang [1], Seunghun Han [3], Taejun Gu [1], Heeyeop Chae [2,3]*, and Dongmok Whang [1,2]*

[1] School of Advanced Materials Science and Engineering, Sungkyunkwan University (SKKU), 2066 Seobu-ro, Jangan-gu, Suwon-si 16419, Republic of Korea; wid0129@skku.edu (W.J.), gtj1008@skku.edu (T.G.)
[2] SKKU Advanced Institute of Nano Technology (SAINT), Sungkyunkwan University (SKKU), 2066 Seobu-ro, Jangan-gu, Suwon-si 16419, Republic of Korea
[3] School of Chemical Engineering, Sungkyunkwan University (SKKU), 2066 Seobu-ro, Jangan-gu, Suwon-si 16419, Republic of Korea; hsh12040@skku.edu (S.H.)
* Correspondence: dwhang@skku.edu (D. W.), hchae@skku.edu (H.C.)

Citation: Jang, W.; Han, S.; Gu, T.; Chae, H.; Whang, D. . hBN Flake Embedded Al_2O_3 Thin Film for Flexible Moisture Barrier. *Materials* **2021**, *14*, 7373. https://doi.org/10.3390/ma14237373

Academic Editors: Filippo Giannazzo and Ivan Shtepliuk

Received: 25 October 2021
Accepted: 29 November 2021
Published: 1 December 2021

Publisher's Note: MDPI stays neutral with regard to jurisdictional claims in published maps and institutional affiliations.

Copyright: © 2021 by the authors. Submitted for possible open access publication under the terms and conditions of the Creative Commons Attribution (CC BY) license (https://creativecommons.org/licenses/by/4.0/).

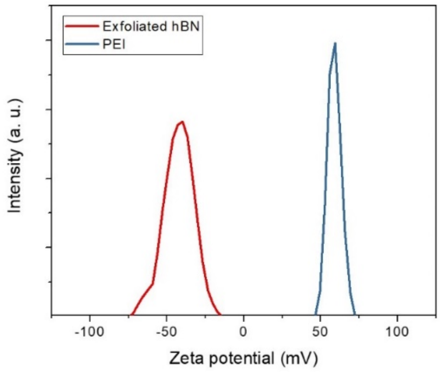

Figure S1. The zeta potential graph of the solutions of PEI polymer and hBN flakes..

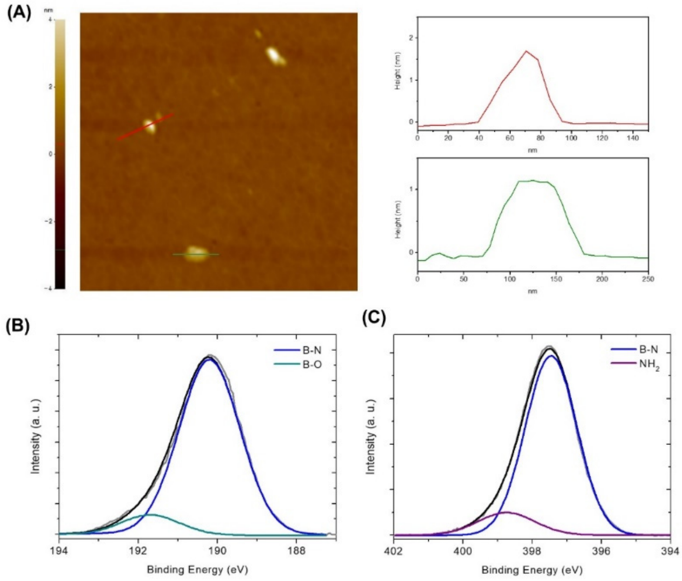

Figure S2. (A) AFM image and height profile of hBN flakes. (B) B 1s (C) N 1s XPS spectra of exfoliated hBN flake

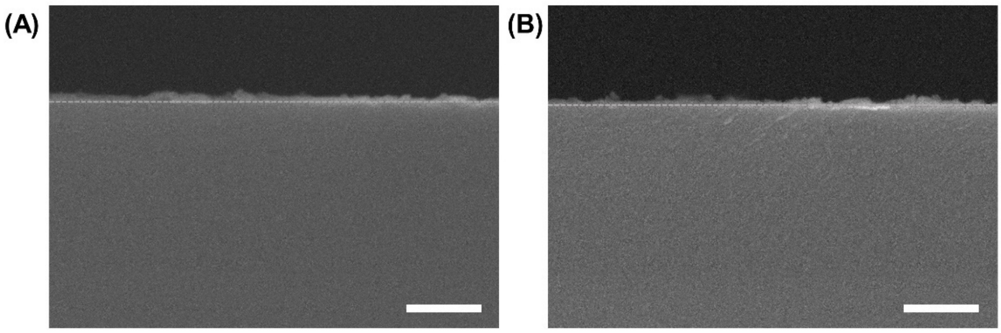

Figure S3. (A) A cross-sectional SEM image of hBN/PEI composite layer. (B) A cross-sectional SEM image showing the intermediate state of Al_2O_3 deposition in hBN/PEI template layer after 100 cycles of ALD deposition in hBN/PEI template layer. The dashed line indicates the interface between the PEN substrate and the composite layer. Scale bars are 100 nm.

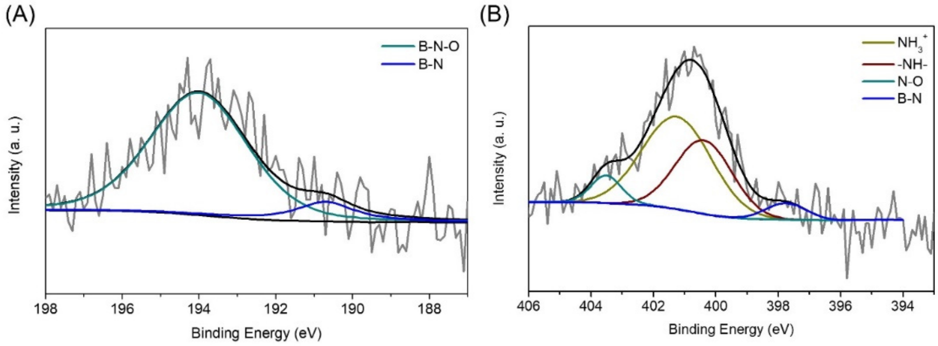

Figure S4. (A) B 1s (B) N 1s XPS spectra of LBL Al_2O_3

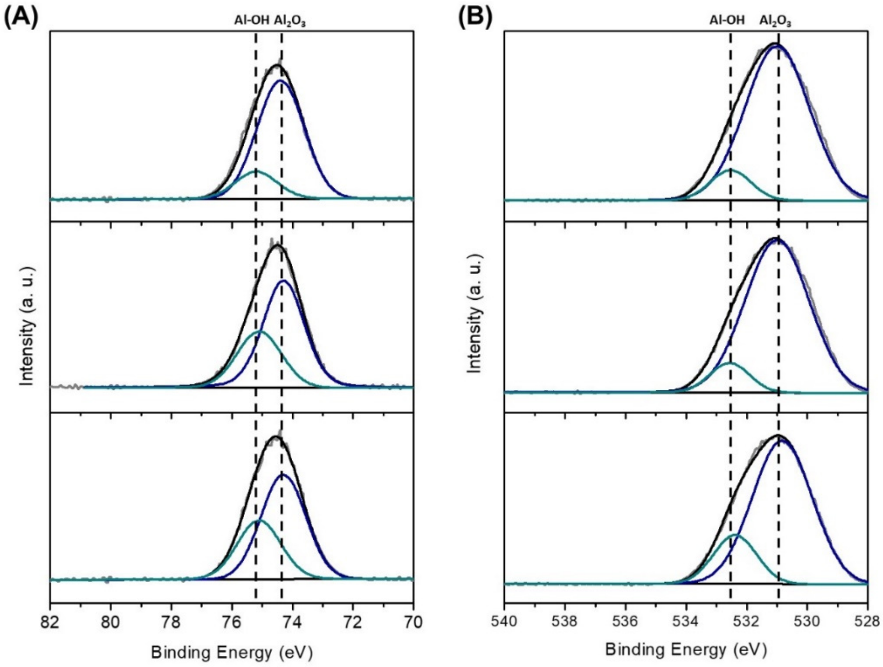

Figure S5. (A) Al 1s (B) O 1s XPS spectra of bare substrate Al_2O_3

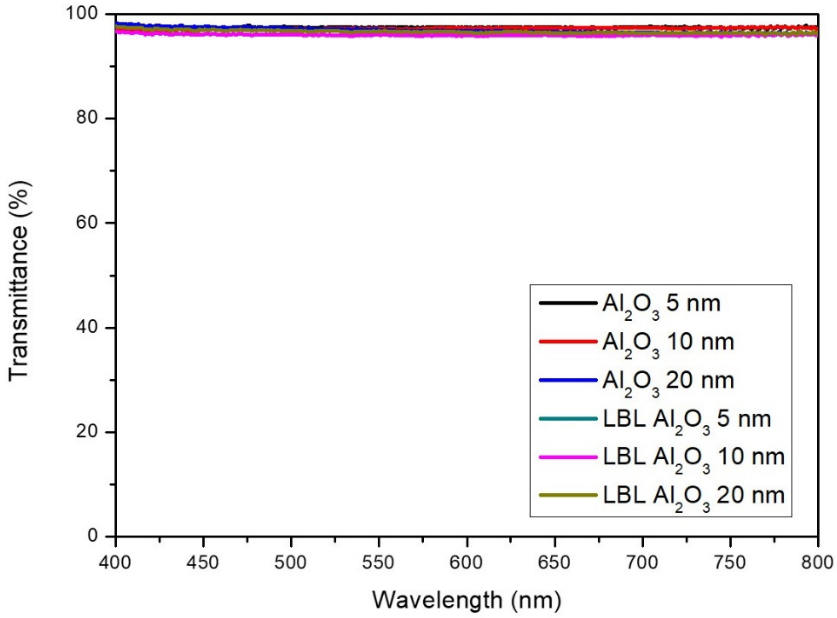

Figure S6. UV-Vis spectra of bare substrate and LBL Al_2O_3 films.

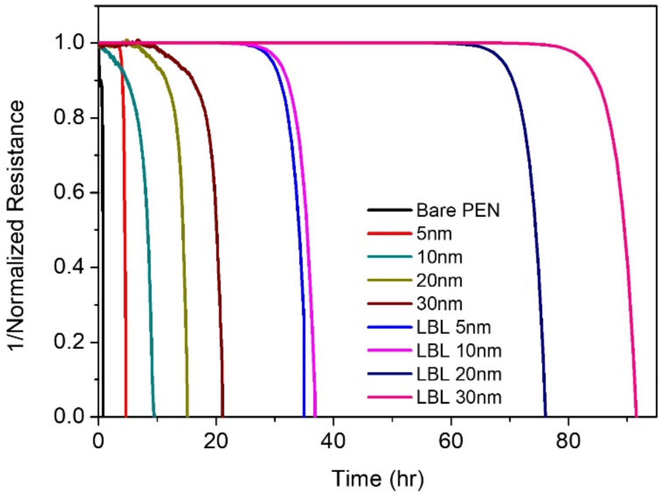

Figure S7. Representative normalized conductance vs. time for Al_2O_3 layers deposited on various substrates and various thicknesses.

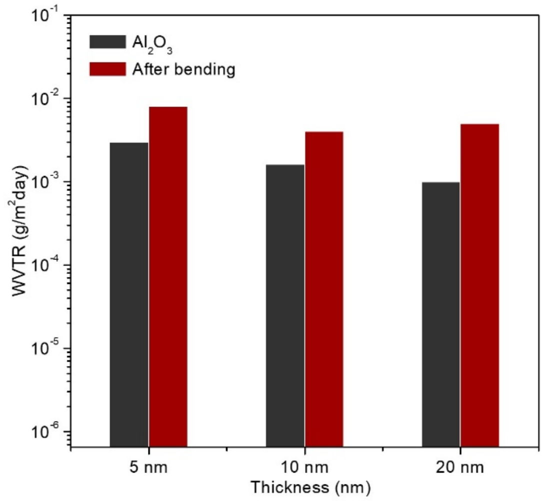

Figure S8. WVTR value of bare substrate Al_2O_3 after outer bending fatigue test (Bending radius : 3 mm, 5000 cycles).
